# Implementation of a comprehensive set of optimised CBCT protocols and validation through imaging quality and dose audit

**DOI:** 10.1259/bjr.20220070

**Published:** 2022-09-01

**Authors:** Marina Khan, Navneet Sandhu, Marium Naeem, Rebecca Ealden, Michael Pearson, Abdirzak Ali, Ian Honey, Amanda Webster, David Eaton, Georgios Ntentas

**Affiliations:** 1 Department of Radiotherapy, Guy’s & St Thomas’ NHS Foundation Trust, London, UK; 2 Department of Medical Physics, Guy’s & St Thomas’ NHS Foundation Trust, London, UK; 3 Department of Radiotherapy, University College Hospital, London, UK; 4 School of Biomedical Engineering and Imaging Sciences, King’s College London, London, UK; 5 Nuffield Department of Population Health, University of Oxford, Oxford, United Kingdom

## Abstract

**Objectives:**

Cone-beam computed tomography (CBCT) for radiotherapy treatment verification has increased in frequency; therefore, it is crucial to optimise image quality and radiation dose to patients. The aim of this study was to implement optimised CBCT protocols for the Varian TrueBeams for most tumour sites in adult patients

**Methods:**

A combination of patient size-specific CBCT protocols from the literature and developed in-house was used. Scans taken before and after optimisation were compared by senior radiographers and physicists to evaluate how changes affected image quality and clinical usability for online image registration. The change in dose for each new CBCT protocol was compared to the Varian default. A clinical audit was performed following implementation to evaluate the changes in imaging dose for all patients receiving a CBCT during that period.

**Results:**

Ten CBCT protocols were introduced including head and neck and patient-size-specific thorax and pelvis/abdomen protocols. Scans from 102 patients with images before and after optimisation were assessed, none of the scans showed image quality changes compromising clinical usability and for some image quality was improved. Between November 2020 and June 2021, 1185 patients had CBCTs using the new protocols. The imaging dose was reduced for 52% of patients, remained the same for 37% and increased for 12%.

**Conclusions:**

This study showed that substantial dose reductions and image quality improvements can be achieved with simple changes in the default settings of the Varian TrueBeam CBCT without affecting the radiographers’ confidence in online image registration.

**Advances in knowledge:**

This study represents a comprehensive assessment and optimisation of CBCT protocols for most sites, validated on a large cohort of patients.

## Introduction

The utilisation of daily cone-beam computed tomography (CBCT) for radiotherapy treatment verification has increased due to the wider application of image-guided radiotherapy (IGRT) and expansion in dose escalation, hypofractionation and reduced margins. Linear accelerators come with default scanning parameters for their CBCT devices which are not always optimised in terms of radiation dose and/or image quality. This can lead to exposing patients to unnecessary amounts of radiation imaging dose or provide suboptimal image quality during treatment, which can lead to image verification uncertainties and repeated scans. Imaging dose from high-dose CBCT protocols over a long course of treatment can deliver a dose equivalent to one fraction of the therapeutic dose, therefore, it cannot be ignored.^1^

CBCT protocols should be optimised to deliver the lowest possible imaging dose whilst maintaining image quality for accurate verification.^2^ Previous studies have suggested methodologies to optimise CBCT imaging parameters based on patient size^3,4^ radiotherapy technique or tumour location.^5–8^ These studies were limited to specific tumour sites (*e.g.,* pelvis) and most reported image quality outcomes from phantom measurements without details on how the optimised images affected the online image registration performed by therapeutic radiographers during treatment.

The purpose of this study was to implement optimised CBCT protocols for most tumour sites for the Varian TrueBeam (Varian Medical Systems, Palo Alto, CA). The aim was to reduce the imaging dose where possible whilst maintaining treatment verification accuracy, or increase dose if necessary to increase image quality. Additionally, to perform a radiographer-led assessment on the image quality of the new CBCT protocols on patient data and complete a retrospective audit on the effect of implementing the new protocols reviewing image quality and dose.

## Methods

A service development proposal to optimise the CBCT protocols was submitted to the local Radiotherapy Operational Group and was approved.

### Optimising CBCT scanning parameters

A literature review was performed to identify studies reporting radiation dose and image quality optimised scanning parameters specifically for Varian Truebeam CBCT. This included milliAmperes (mA), scanner exposure time per rotation (mS), and number or projections and acquisition time. The CBCT anatomical sites included head and neck (H&N), thoracic (Thorax), and abdominal and pelvis (Abdo/Pelvis). The utilisation of patient size-specific scanning settings was explored for thorax and Abdo/pelvis.

Using optimised protocols from both published literature and developed in-house, new CBCT protocols were introduced for all tumour sites (Table 1). For H&N treatments, protocol settings from two published studies were used, a lower dose protocol by Agnew et al^4^ for conventional radiotherapy (*e.g.,* VMAT) and a higher dose protocol by Mao et al^5^ for stereotactic radiosurgery (SRS) treatments. For Thorax and Abdo/Pelvis CBCTs, dose-optimised patient-size specific protocols previously proposed by Agnew et al^4^ were used. The size selection methodology was based on the average patient diameter which is calculated as:

Average Diameter = (max lateral separation+max anteroposterior separation)/2

**Table 1.** Patient size-specific scanning settings, measured imaging doses and change in dose for the new protocols compared to the Varian default protocols

CBCT Protocols	kV	mA	ms	f/s	Gantry speed (^o^/S)	Trajectory	Number of projections	mAs	CTDI in Air (mGy)	CTDI_w_ (mGy)^a^	Change in CTDI_air_ (%)
**Varian_Head**	100	14	20	15	6	Half	500	140	6.2	3.1	n/a
**Head_Standard**	100	10	15	15	6	Half	500	75	3.1	1.8	−50%^b^
**SRS_Head**	100	30	20	15	6	Half	500	300	10.5	6.2	70%^b^
**Varian_Thorax**	125	15	20	15	6	Full	900	270	18.3	5.0	n/a
**Thorax_S**	125	10	10	15	6	Full	900	90	7.9	2.0	−57%^c^
**Thorax_M**	125	15	15	15	6	Full	900	202.5	14.6	3.9	−20%^c^
**Thorax_L**	125	25	15	15	6	Full	900	337.5	22.3	5.9	22%^c^
**Varian_Abdo/Pelvis**	125	38	20	15	6	Full	900	684	42.8	11.5	n/a
**Abdo/Pelvis_S**	125	25	10	15	6	Full	900	225	15.7	4.2	−63%^d^
**Abdo/Pelvis_M**	125	38	20	15	6	Full	900	684	42.8	11.5	0%^d^
**Abdo/Pelvis_L**	125	60	20	15	6	Full	900	1080	66.3	17.9	55%^d^
**Abdo/Pelvis_HD**	125	38	20	15	3	Full	1800	1368	85.6	22.8	100%^d^
**Bariatric**	125	100	25	15	6	Full	900	2250	130.0	36.7	n/a

Size small<26 cm (S), medium>26 cm <36 cm (M), Large>36 cm (L) average diameter.

a CTDI_w_ measurements were taken in the centre and four peripheral points of the TO CTDI phantom (Leeds Test Objects) to calculate weighted CTDI values (CTDIw). For the head protocols the 160 mm cylindrical object was used and for the thorax and abdo/pelvis the 320 mm cylindrical object. Note these should be considered as indicative values, not a true CTDIw, due to the difference in geometry and scatter condition between CBCT and CT.

b Change versus Varian Default protocol (Varian_Head)

c Change versus Varian Default protocol (Varian_Thorax)

d Change versus Varian Default protocol (Varian_Pelvis)

Based on their average diameter (AD), patients were assigned a Small (S), Medium (M), or Large (L) scan if AD≤26 cm, 26cm > AD<36 cm or AD≥36 cm, respectively.

**Figure 1.**
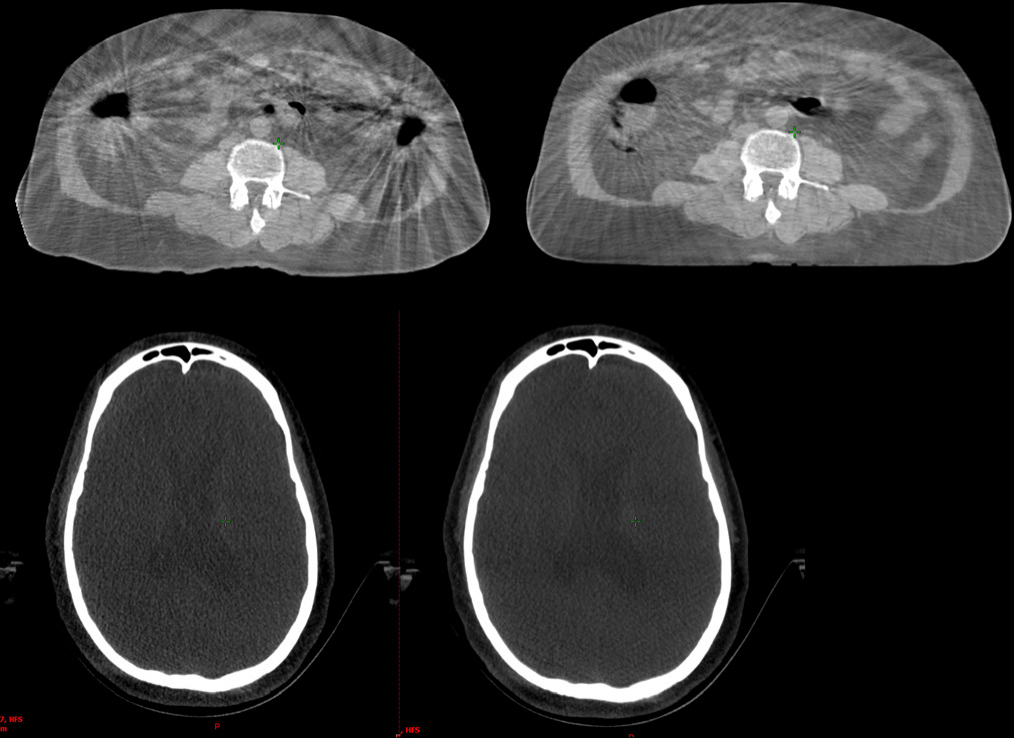
Abdomen scan taken with Varian default pelvis CBCT protocol (top left) and scan taken with Abdo/Pelvis_HD (top right). Head scan taken with Varian default Head CBCT protocol (bottom left) and scan taken with Head_SRS (bottom right). Both Abdo/Pelvis_HD and Head_SRS increased the imaging dose and improved image quality and the radiographers’ confidence to perform online image registration.

A “high-definition“ protocol (Abdo/Pelvis_HD) was developed in-house for patients whose abdominal CBCT image quality was severely affected by lack of soft tissue definition and artefacts as a result of gas and internal motion which can be seen in Figure 1. The gantry rotation speed was reduced (from 6 to 3 degrees/S), which resulted in an increase of the number of projections per full rotation (from 900 to 1800). This was based on the hypothesis that acquiring more images per rotation would decrease artefacts due to the expected improvement in contrast-based image quality metrics.^9^ Finally, an Abdo/Pelvis protocol (bariatric) of increased mAs was developed in-house for bariatric patients for whom the large Abdo/Pelvis protocol images will be too noisy to visualise the target and for accurate online image registration.

### Testing phase

CT dose index (CTDI) in-air (mGy) measurements were performed using the RaySafe^TM^-X2-CT Sensor. Additionally, CTDI measurements were taken in the centre and four peripheral points of the TO CTDI phantom (Leeds Test Objects) to calculate weighted CTDI volume values (CTDIw). For the head protocols, the 160-mm cylindrical object was used and for the thorax and abdo/pelvis protocols, the 320-mm cylindrical object was used with the 160-mm object nested inside, again using the RaySafe^TM^-X2-CT Sensor. The measurements were taken for both the new and the previously utilised Varian default protocols to compare dose differences (Table 1). Following the radiation dose assurance measurements, the new CBCT protocols were installed in two out of eight linacs in the department and utilised to scan patients. These patients were on treatment at that time and had images acquired with the default Varian protocols up to the day before installing the new protocols. The images from the new protocols would be compared to the previous days acquired with Varian’s default protocols. All optimised protocols were tested comprising the “Testing Group”, except for the bariatric protocol as a patient of that size was not having treatment at the time.

Image quality and clinical usability of the new CBCTs were reviewed for all patients in the Testing Group both online and offline by two different teams. Each team included two senior radiographers and a physicist (online and the offline team). Clinical usability was defined as the CBCT scan being at least as clinically acceptable for accurate 3D-image registration to the planning CT data set or better than the scans taken with the Varian default protocols. This was first tested online using the automatic registration software on the True Beam On-Board Imaging (OBI) system. A standard pre-defined region of interest was set to test if the online auto-registration between the planning CT and CBCT images performed as well as the day before, when the CBCT images from the non-optimised protocols were used. A manual image registration was also performed by the same team (online team) of senior radiographers and physicist to assess the ease and accuracy of the image registration visually. The other team (offline team) assessed the image registration offline to ensure that the target volume was adequately visible in the Elekta Mosaiq (Elekta AB, Stockholm, Sweden) image review workspace. The offline review was also a radiographer-led qualitative comparison replicating the same criteria and scoring system utilised during the online review to compare the CBCT images before and after optimisation and it is described in Table 2. The offline review was undertaken by different radiographers and physicist to those who performed the online review to avoid bias and test for interobserver variability. One score per protocol was given by each team (online and offline) to be representative of the clinical situation during the treatment when a team assesses the image registration rather than one individual (Table 2).

**Table 2.** Scoring system for comparing CBCT scans before and after optimisation

Score	Image quality and usability for image registration	Testing Group (n)		Implementation Group (n)
		Online review	Offline review	Offline review
A	Improved image quality and image registration	2	2	4
B	Improved image quality no change in image registration	1	1	2
C	No change in image quality and image registration	2	2	71
D	Slightly lower image quality but no change in image registration	4	4	16
E	Lower image quality and compromised image registration	0	0	0
**Total:**		**9**	**9**	**93**

### Implementation

Following the testing phase, the new CBCT protocols were installed on all eight linacs. All patients treated at the time of implementation, and who therefore would have received a CBCT with both the default and optimised settings, comprised the “Implementation Group”. A qualitative comparison between the pre- and post-optimisation CBCTs was performed offline for all patients in the Implementation Group using the scoring criteria by two senior radiographers (Table 2). The radiographers were blind to the protocol changes to avoid bias and to ensure the initial findings during the testing phase were confirmed independently.

## Clinical audit

An audit was performed seven months following implementation to evaluate the changes in imaging dose for all patients receiving a CBCT during that period. Information about the CBCT protocols was collated for all patients who received CBCTs between November 2020 and June 2021.

## Results

### Optimised CBCT protocols

Ten new CBCT protocols were introduced, covering most sites treated in the department (Table 1). Relative dose differences (CTDIair and CTDIw) are shown in Table 1; the dose differences described below are based on CTDIair. Two head and neck protocols were created: a “Head_Standard” for patients requiring head CBCTs for conventional VMAT radiotherapy, used predominantly in H&N and brain cancer patients, which reduced the imaging dose by 50% compared to the Varian default settings and a “Head_SRS”, higher dose protocol for patients undergoing intracranial SRS radiotherapy to improve soft-tissue visualisation. The latter doubled the dose compared to the Varian default settings. For thoracic CBCTs, which are used predominantly in lung, upper gastrointestinal (GI), lymphoma and breast cancer patients, the S protocol reduced the imaging dose by 57%, the M protocol by 20% whilst the L protocol increased the dose by 22% compared to the Varian default. For Abdo/Pelvis CBCTs, which are used predominately in prostate, gynaecological, hepatobiliary and upper/lower GI cancer patients, the S protocol reduced the dose by 63%, the dose for the M protocol was the same as the Varian default whilst the L protocol increased the dose by 55%. The dose from the Abdo/Pelvis_HD was doubled compared to Abdo/Pelvis M protocol which is due to the double number of projections taken per rotation. The dose from the “Bariatric” protocol was almost double that to Abdo/Pelvis L (Table 1).

## Testing

**Figure 2.**
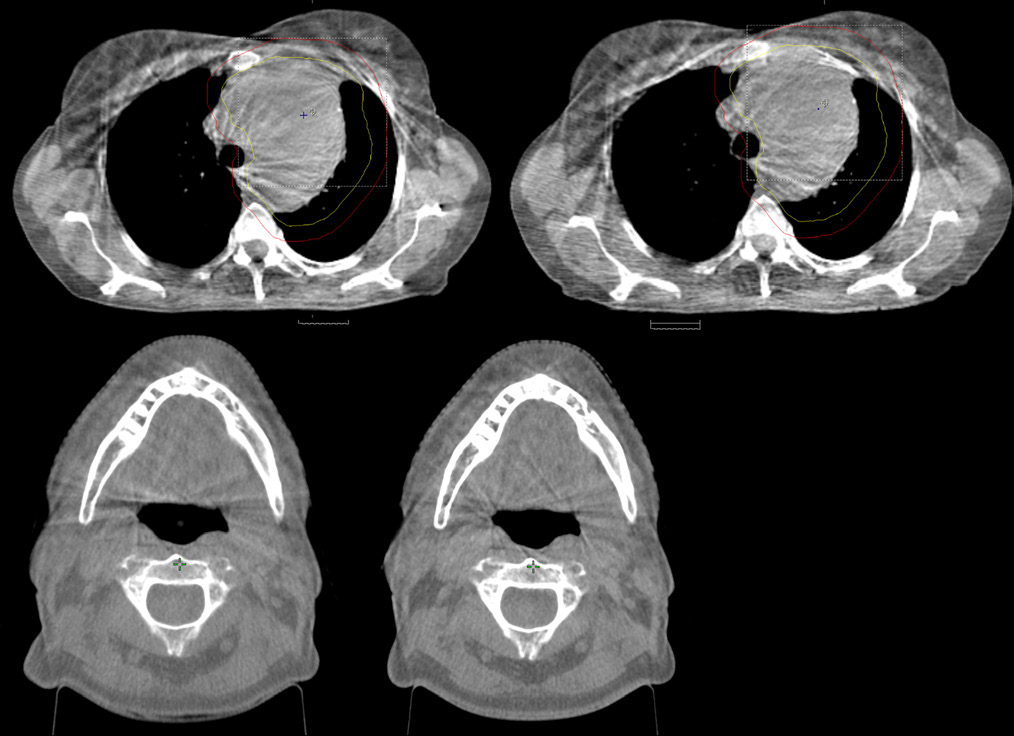
Thoracic scan taken with Varian default pelvis CBCT protocol (top left) and scan taken with Thorax_S (top right). Head scan taken with Varian default Head CBCT protocol (bottom left) and scan taken with Head_Standard (bottom right). Both Thorax_S and Head_Standard decreased the imaging dose without affecting the radiographers’ confidence to perform online image registration.

The lower dose protocols did not reduce the image quality enough to affect the online image registration (Table 2). Both bone and soft tissue were visible enough for the TrueBeam auto-registration software to perform accurate 3D-image registration and the radiographers were also able to perform manual registration. The offline review of all images for the Testing Group confirmed these findings. Four out of nine scans showed slight reduction in image quality compared to those taken with the Varian default protocol the previous day but was negligible and did not affect online image registration (Score D). Two examples are given for Standard_Head and Thorax_M in Figure 2. Two of the nine scans showed no change in image quality (Score C) and one of the nine showed improved image quality but without improving confidence in online image registration (Score B). Finally, it was found two of the nine acquired images (SRS_Head and Abdo/Pelvis_HD) improved image quality and radiographers’ confidence to perform online registration (Score A) as can be seen in Figure 1.

## Implementation

**Figure 3.**
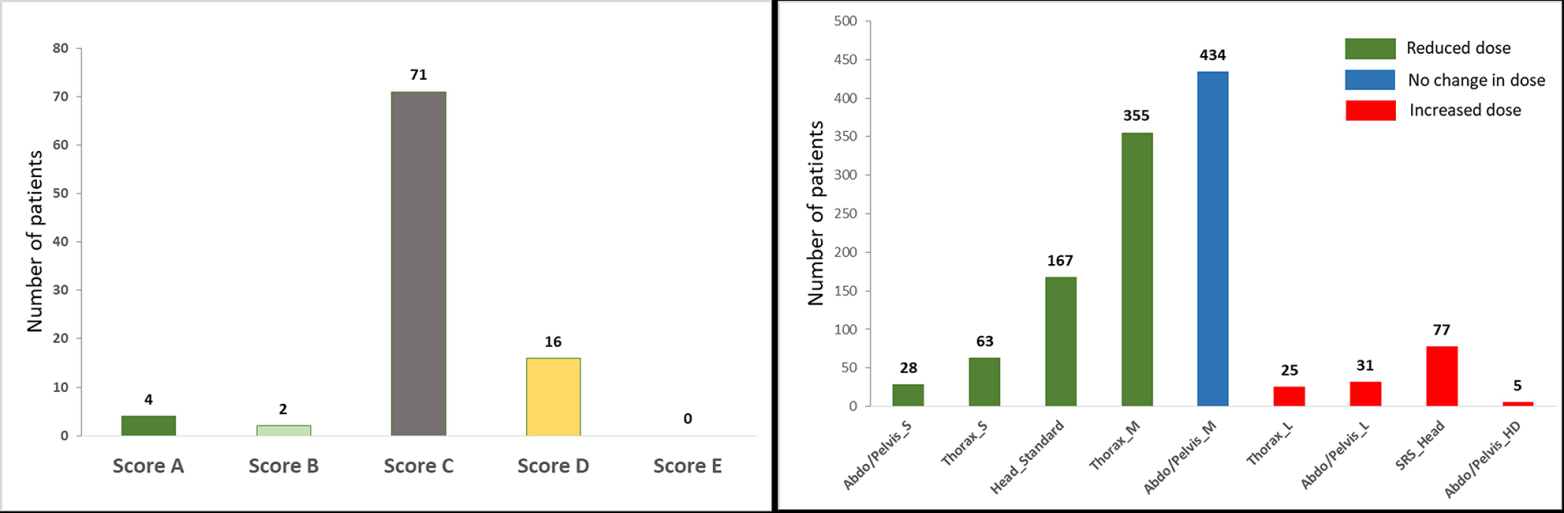
Patients in the Implementation Group distributed by image quality score* achieved (Left). Patients in the Clinical Audit Group distributed by dose change per CBCT protocol when compared to the Varian default settings (Right).*Scoring - A: Improved image quality and image registration, B: Improved image quality no change in image registration, C: No change in image quality and image registration, D: Slightly lower image quality but no change in image registration, E: Lower image quality and compromised image registration.

The results from the Implementation Group confirmed the findings of the Testing Group above. 93 patients who had CBCT images taken before and after the implementation of the new protocols were identified (Tables 2 and 3). None of the new protocols produced an image with inferior quality enough to affect image registration compared to the Varian default scans. All patients who had a CBCT scan using Head_standard, Thorax_S, Thorax_M or Abdo/Pelvis S, and for whom the imaging dose was reduced, were scored as C or D, i.e. the image quality was the same or slightly inferior, and the dose reduction did not affect the radiographers’ confidence to assess the automatic registration and furthermore visualisation of the target volume, similar to the example in Figure 2. Patients, who had a CBCT scan using Thorax_L or Abdo/Pelvis_L, and for whom the imaging dose was increased, were scored as B or C, apart from one patient who had a Thorax_L scan but was scored D, i.e. the dose increase did not improve the radiographers’ confidence during image registration. All patients who had CBCT scans using SRS_Head or Abdo/Pelvis_HD were scored A, i.e. the dose increase improved image quality and confidence in image registration. (Figure 1). The left graph in Figure 3 shows the distribution of the patients in the Implementation Group based on the image quality scoring. Detailed scoring for all 93 patients can be seen in Supplementary Material 1.

Supplementary Material 1.

**Table 3.** Number of patients per CBCT protocol for the three patient groups and change in imaging dose compared to the Varian default protocols

			Patient number		
**Protocol**	**%Change in imaging dose^a^**	**Dose change (mGy)^a^**	**Testing Group (n**)	**Implementation Group (n**)	**Clinical Audit Group** (**n**)
Head_standard	−50%^b^	−3.1	1	8	167
SRS_Head	70%^b^	4.3	1	1	77
Thorax_S	−57%^c^	−10.4	1	2	63
Thorax_M	−20%^c^	−3.7	1	18	355
Thorax_L	22%^c^	4.0	1	4	25
Abdo/Pelvis_S	−63%^d^	−27.1	1	1	28
Abdo/Pelvis_M	0%^d^	42.8	1	53	434
Abdo/Pelvis_L	55%^d^	23.5	1	3	31
Abdo/Pelvis_HD	100%^d^	42.8	1	3	5
**Total patient number**			**9**	**93**	**1185**

a Compared to Varian Default settings.

b Change versus Varian Default settings for Head CBCT as can be seen in Supplementary Material 1

c Change versus Varian Default settings for Thorax CBCT as can be seen in Supplementary Material 1

d Change versus Varian Default settings for Pelvis CBCT as can be seen in Supplementary Material 1

## Clinical audit

Between November 2020 and June 2021, 1185 patients had CBCTs using the optimised protocols (Clinical Audit Group). The imaging dose was reduced for 52% of patients (613 out of 1185), it remained the same for 37% (434) and was increased for 12% (138). A breakdown of patient numbers for each protocol can be seen on Table 3 and Figure 3. Compared to patients having had a CBCT using the Varian default settings, the imaging dose was reduced by 63% (from 42.8 to 15.7 mGy) for 28 patients who were scanned with the Abdo/Pelvis_S, by 57% (from 18.3 to 7.9 mGy) for 63 patients scanned with the Thorax_S, by 50% (from 6.2 to 3.1 mGy) for 167 patients scanned with the Head_Standard and by 20% (from 18.3 to 14.6 mGy) for 355 patients scanned with the Thorax_M protocol. On the contrary, the dose was increased by 70% (from 6.2 to 10.5 mGy) for 77 patients using the SRS_Head protocol and by 55% (from 42.8 to 66.3 mGy) for 31 patients scanned with the Abdo/Pelvis_L. Five patients were scanned with Abdo/Pelvis_HD (from 42.8 to 85.6 mGy). No patient was scanned with the Bariatric protocol and the dose remained the same for 434 patients scanned with the Abdo/Pelvis_M protocol.

## Discussion

In this study, implementation of a comprehensive set of optimised CBCT protocols and radiographer-led validation through imaging quality and dose audit was performed for most tumour sites. The image quality changes and effect on online image registration was evaluated for 93 patients (Implementation Group) and potential clinical benefit (imaging dose reduction) was investigated in a large cohort of 1,278 patients (Implementation and Clinical Audit Groups). It was demonstrated that the optimised CBCT protocols reduced the imaging dose delivered during on-treatment imaging for most patients (52% of patients) without affecting the radiographers’ confidence in image registration. A patient size-specific selection method for dose optimisation in thoracic, abdominal and pelvic CBCT protocols was successfully implemented in a large radiotherapy department without requiring additional time resources or staffing and without delaying the patient pathway. This study confirms that substantial imaging dose reductions can be achieved with simple changes in the default settings of the Varian TrueBeam CBCT for most sites. Finally, it has been shown that in some cases when more accurate image registration is required, for example SRS treatments, increasing the dose can lead to improved image quality and more confident online image registration. The CBCT protocols proposed in this study can be used by any radiotherapy centre with Varian TrueBeam Linacs that are currently using the default Varian CBCT settings.

The patient size-specific protocol selection for Thoracic and Abdo/Pelvis imaging was easily incorporated in the treatment verification procedures. The S and the M thorax protocols provided the biggest dose reductions without affecting image quality enough to compromise image registration as it can be seen in Figure 2. The Thorax_L and Abdo/Pelvis_L protocols improved the image quality marginally but the radiographers’ evaluating the registration did not observe a substantial improvement as the three patients evaluated (one in Testing and two in implementation groups) scored as “B- Improved image quality no change in online match”, therefore, it was decided to re-evaluate its usefulness on more patients in the future.

For a small group of patients who have gone through abdominal surgery and have surgical clips or high-density implants, artefacts can affect the radiographers’ ability to visualise the tumour, OAR’s and perform image registration. Increasing the mAs did not improve the image quality therefore an Abdo/Pelvis_HD protocol was created by slowing down the gantry rotation speed, acquiring more projections, which helped improve the image quality and the radiographers’ confidence to visualise the target and the OARs (Figure 1). A 2 to 3 ^0^/S gantry speed, the one used for the Abdo/Pelvis_HD protocol in this study has been proposed as optimal to improve contrast-based image quality metrics (*e.g.,* spatial resolution and low contrast detectability) and to reduce image noise and artefacts in the Varian CBCT scans.^9^

Two protocols were introduced for patients requiring head and neck CBCT imaging. Head_standard is used for conventional fractionation for all cancers in the H&N region and due to the high number of fractions the dose reduction benefit for these patients is substantial. For SRS treatments, where high doses are delivered in a single fraction with smaller targets and margins, increasing the image quality is crucial. The increased image quality allowed better soft tissue contrast and tumour visualisation which is important for these types of treatments to ensure radiographers can visualise the multiple targets and perform accurate image registration. The increased imaging radiation dose in these cases is justified as these patients receive one fraction or a maximum of five fractions.^5^

Ordonez-Sanz et al^7^ proposed a different approach for optimising CBCT using individual patient attenuation from the patients planning CT (CTDI values) for the Clinacs and TruBeam Varian Linacs. They used lower kV (100kV instead of 125kV) for their S and M pelvic protocols but higher mAs compared to the protocols in this study and their CTDIs measured in air were comparable to this study. However, they did not provide information on thoracic or head and neck protocols. We chose to utilise the patient size selection methodology proposed by Agnew et al as it is focused on the treated area and thus where the CBCT images will be taken. Additionally, the authors derived their results on a representative patient cohort and validated usability with radiographers.

## Strengths and limitations

In this study, a comprehensive implementation of optimised CBCT protocols was performed for most tumour sites by considering the type of radiotherapy delivered and fractionation regime. One of the main strengths was the validation of these protocols via direct comparison of images before and after optimisation on 93 patients (Implementation Group), where data showed comparable image quality for all patients even after reducing radiation dose by up to 63%. Additionally, the reporting on clinical data from a large cohort of 1,185 patients (Clinical Audit Group) increase confidence that a substantial number of patients will receive lower imaging dose compared to the manufacturer’s default settings if our proposed protocols are installed.

We have independently validated suggested protocols from other authors/centres and found that protocol changes can be easily implemented at any radiotherapy department using the Varian True beam Linacs. A pragmatic radiographer-led clinical approach in assessing the change in image quality before and after optimisation was used to ensure that the changes were clinically relevant and impactful. Additionally, a novel approach to reduce the gantry speed to acquire more projections per rotation to reduce gas and metal artefacts from surgical clips in pelvic and abdominal scans was implemented and validated on patients. Whilst this methodology did improve image quality in these cases with extreme artefacts, this protocol should be used with caution as the clinical benefit from the improved image quality should be justified with the increased dose for each patient individually. One of the limitations in this study is that the proposed CBCT protocol settings are only directly applicable to Varian TrueBeam Linacs. However, centres with different Linacs from a different manufacturer can still adopt our methodology. Additionally, whilst a radiographer-led qualitative evaluation of image quality is crucial to ensure clinically meaningful changes, it can also be subjective and prone to bias. We used independent groups of radiographers to evaluate the images blind to protocol changes to reduce bias. Another limitation in our study is that we provide CTDI measured in air which is not appropriate for accurate absolute dose evaluation due to its inability to accommodate and record the whole primary beam and scattered radiation.^10^ Additionally, CTDIvol measurements whilst well defined for diagnostic CTs, are not well defined for CBCT where the scanning and scatter conditions, geometry, beam quality and beam shaping are very different to a diagnostic CT. The use of a standard CTDI measurement phantom like the one we used underestimates CTDIvol and consequently the patient dose as part of the wide CBCT beam would miss the phantom entirely leading to an absence of scatter reaching the chamber from these peripheral regions. Consequently, we did not estimate effective doses as any method of calculating effective dose from CBCT would rely on these CTDIvol measurements which we did not have confidence in for the reasons above. Additionally, available effective dose calculation methodologies would be based on CT rather than CBCT geometries, adding further to the uncertainties. Further research is needed in that field to establish national levels and a recommended methodology to estimate effective doses for each CBCT protocol. However, this study aimed to report relative dose reductions between protocols which can still be fully assessed using CTDI measurements.

The rapid changes in radiotherapy technologies, planning techniques and fractionation regimes require frequent re-evaluation of imaging scanning parameters such as mAs, KV energy, and gantry speed, frequency of scans, justification process and clinical audits. If, for example, the fractionation regimes of specific tumour sites change substantially, the CBCT protocols used for this site should be re-evaluated to ensure imaging protocols provide optimal imaging to our patients whilst reflecting the continuously changing radiotherapy landscape.
