# Supplementary material for: Implementation of a comprehensive set of optimised CBCT protocols and validation through imaging quality and dose audit
Source: Br J Radiol. 2022 Sep 1;95(1139):20220070. doi: 10.1259/bjr.20220070 (PMC9793481; doi:10.1259/bjr.20220070)
Supplement: Supplementary Material 1. [file bjr.20220070.suppl-01.docx]

**Supplementary Materials:** “Implementation of a comprehensive set of optimised CBCT protocols and validation through imaging quality and dose audit”

Supplemental Table 1. Scoring of image quality for CBCT scans before and after optimisation for patients in the Implementation Group (n=93).

| CBCT Protocol | Total number of scans | Change |
| --- | --- | --- |
| Abdo/Pelvis_S | 1 | D |
| Abdo/Pelvis_M | 1 | D |
| Abdo/Pelvis_M | 52 | C |
| Abdo/Pelvis_L | 3 | C |
| Abdo/Pelvis_L | 2 | B |
| Abdo/Pelvis_HD | 3 | A |
| Thorax_S | 2 | D |
| Thorax_M | 13 | C |
| Thorax_M | 5 | D |
| Thorax_L | 1 | C |
| Thorax_L | 1 | D |
| Head_standard | 6 | D |
| Head_standard | 2 | C |
| SRS_Head | 1 | A |
